# Potential Material Basis of Yupingfeng Powder for the Prevention and Treatment of 2019 Novel Coronavirus Pneumonia: A Study Involving Molecular Docking and Molecular Dynamic Simulation Technology

**DOI:** 10.1155/2022/7892397

**Published:** 2022-06-24

**Authors:** Ying Yu, Gong Zhang, Tao Han, Hongjie Liu, Hailiang Huang

**Affiliations:** ^1^Doctor's Degree of Medicine, In Station Post-Doctorate, Innovative Institute of Chinese Medicine and Pharmacy, Shandong University of Traditional Chinese Medicine, Jinan 250355, China; ^2^Doctor's Degree of Medicine, College of Integrated Traditional Chinese and Western Medicine, Shandong Liming Vocational College of Science and Technology, Tai'an 271000, China; ^3^Doctor's Degree of Medicine, Graduate Office of Shandong University of Traditional Chinese Medicine, Jinan 250355, China; ^4^Doctor's Degree of Medicine, School of Traditional Chinese Medicine, Jinan University, Guangzhou 510632, China; ^5^Doctor's Degree of Medicine, College of Rehabilitation Medicine, Shandong University of Traditional Chinese Medicine, Jinan 250355, China

## Abstract

**Objective:**

In this study, we investigated the potential material basis of Yupingfeng powder in the prevention and treatment of 2019 novel coronavirus pneumonia (NCP) by applying molecular docking and molecular dynamic simulation technology.

**Design:**

The active ingredients and predictive targets of Yupingfeng powder were sourced using the TCMSP, ETCM, and TCMIP traditional Chinese medicine databases. NCP-related targets were then acquired from the DisGeNET and GeneCards databases, and common disease-drug targets were imported into the STRING database, and Cytoscape software was used to generate a protein-protein interaction network following the use of a network topology algorithm to identify key target genes. Gene Ontology (GO) and KEGG pathway enrichment analysis was then performed using the target genes and GOEAST and DAVID online tools. The mechanism of Yupingfeng powder in the prevention and treatment of NCP was analyzed with reference to the relevant literature. AutoDock software was used for molecular docking, the preliminary analysis of binding status, and to identify the best conformation. Desmond software was used to perform molecular dynamic simulations for protein and compound complexes, perform free energy calculations and hydrogen bond analysis, and to further verify the binding mode.

**Results:**

Overall, 38 main active components and 218 predictive targets of Yupingfeng powder were identified and 298 disease targets related to NCP were retrieved from disease databases. Yupingfeng powder was found to act predominantly on the TNF, Toll-like receptor, HIF-1, NOD-like receptor, cytokine-receptor interaction, MAPK, T cell receptor, and VEGF signaling pathways. Molecular docking of the three selected key active components with the 3CL-like protease (3CL-Pro) of SARS-CoV-2 showed that they each had a strong binding force and good affinity.

**Conclusions:**

Yupingfeng powder primarily acts on multiple active ingredients and potential targets through multiple action channels and signal pathways. Molecular docking and molecular dynamic simulation technology were used to effectively predict and analyze the potential mechanism by which this Chinese medicine can combat NCP. These results provide a reference for developing new modern Chinese medicine preparations against NCP in the future.

## 1. Introduction

Novel coronavirus pneumonia (NCP), which the World Health Organization has officially named “coronavirus disease 2019” (COVID-19) [1], is an acute form of infectious pneumonia. The main clinical manifestations in patients are fever, fatigue, and a persistent dry cough. In severe cases, dyspnea (shortness of breath) has been observed to develop within a week, and can, in some instances, rapidly develop into acute respiratory distress syndrome, septic shock, or multiple organ failure, leading to death [2]. The disease is highly infectious and epidemic; the number of new confirmed cases, suspected cases, and mortality rates in most countries and regions is increasing rapidly. Moreover, the daily reports of newly confirmed cases, suspected cases, and deaths around the world have all shown a sharp upward trend. As of the 19^th^ October 2021, the cumulative number of confirmed cases exceeded 241,909,397 worldwide and resulted in outcomes that carries serious consequences for physical and mental health and the quality of life and work; these effects pose significant long-term threats that can exert broad implications on social stability and economic development [3, 4].

The research teams of Zihe Rao and Haitao Yang at Shanghai University of Science and Technology have identified the high-resolution crystal structure (PDB 6LU7) of the 3CL hydrolytic protease of the severe acute respiratory syndrome coronavirus 2 (SARS-CoV-2) virus, thereby providing a basis for screening the active ingredients against the specific viral strain that causes COVID-19 [5, 6]. However, there are many types of respiratory viruses and each can be prone to mutation. As yet, researchers have not been able to elucidate the specific pathogenesis of SARS-CoV-2 and there are no specific treatments available at present. Western medicine is primarily investigated antiviral drugs, such as interferons, lopinavir-ritonavir combinations, and ribavirin, as well as the antimalarial chloroquine phosphate. However, with the prolongation of the medication cycle, patients inevitably experience different degrees of side effects which can reduce compliance and curative effects for patients during the treatment process. Alternatively, traditional Chinese medicine (TCM) offers the distinctive advantages of overall regulation and syndrome differentiation for disease prevention. Not only does TCM have substantial foundation in clinical practice, many classic prescriptions have been generated over time. Furthermore, TCM has historically played a crucial role in the treatment of various respiratory viral diseases and in combating epidemics; consequently, TCM has attracted widespread attention.

In the present study, we reviewed the prescriptions and performed statistical analysis of the prevention schemes of TCM that have demonstrated clinical curative effects in various provinces and cities in China and found that Yupingfeng powder is the basic prescription for clinical prevention in many TCM schemes. The powder consists of *Astragalus* root (*A. membranaceus*; *huangqi* in Chinese), *Atractylodes macrocephala* (*baizhu,* in Chinese), and siler root (*Saposhnikovia divaricata*; *fangfeng* in Chinese). This powder can benefit *qi*, strengthen the surface of the body, and stop perspiration; it has become the classic ancient prescription for strengthening the foundation of life. Modern pharmacological studies have shown that this prescription can play a role in immune regulation and maintain an anti-inflammatory, bacteriostatic, and stable microecological environment. It also exerts antitumor potential, *via*a variety of mechanisms, and has a significant effect on repeated upper-respiratory tract infection, influenza, refractory mycoplasma pneumonia, bronchial asthma, and other respiratory diseases [7, 8].

Given its overall treatment characteristics in relation to multiple targets and the additional and numerous effects of this TCM compound, Yupingfeng powder appears to offer certain advantages and potential for addressing the current pandemic. However, there is a lack of comprehensive and systematic understanding of its active constituents and the molecular mechanisms of action, thus limiting the discovery, application, and promotion of this TCM compound and its preparation for treating NCP. Therefore, in the present study, we identified the key active components and targets of Yupingfeng powder using a combination of molecular docking and molecular dynamic simulation technology. Our aim is to systematically analyze the potential preventive mechanism of the prescription and to further explore potential relationships between the prescription and the disease. Next, we aimed to identify the best matching pattern of drug targets by complementing the spatial structure of active receptor sites. By adopting this approach, we hoped to provide a feasible strategy for basic research, clinical application, and new drug development towards the prevention and treatment of NCP by TCM in the future. Our findings may also advance the promotion of TCM towards globalization, internationalization, and modernization.

## 2. Methods

### 2.1. Screening Active Components and Prediction Targets in Yupingfeng Powder

We used analysis platforms, along with TCMSP, ETCM, TCMIP, and other TCM databases and the names of three herbs (*Astragalus membranaceus*, *Atractylodes macrocephala*, and *Saposhnikovia divaricata*) as keywords to retrieve all chemical composition information for each herb. Following the pharmacokinetic ADME (Absorption, Distribution, Metabolism, and Excretion) parameters recommended in relation to the TCMSP platform, the screening parameters of “oral bioavailability” and “drug likeness” were set at ≥30% and ≥0.18, respectively. Information relating to key active ingredients was collated and combined with herbal compound data obtained from the ETCM and TCMIP databases. A final data list for the compounds was obtained by eliminating any duplicated information. Next, we sourced the target protein information corresponding to the predicted compounds in the database, standardized the target protein name by reference to the UniProt platform, and finally summarized the target data for the compounds as the prediction targets of the main active ingredients of Yupingfeng powder.

### 2.2. Acquisition of a Target for NCP

Using “novel coronavirus pneumonia” as the key search term and “*Homo sapiens*” as the selected species, we searched the DisGeNET and GeneCards disease databases, combined the target genes from each database, deleted duplicated targets, and finally identified NCP-related genes.

### 2.3. Construction of a Database of Intersecting Drug-Disease Target Genes

We imported the target data for herbs and diseases into the OmicShare online tool to generate a Venn diagram, thus mapping the potential target genes of Yupingfeng powder for the prevention and treatment of NCP. Finally, the intersecting target genes were identified.

### 2.4. Construction of a Protein-Protein Interaction Network

We uploaded the intersecting targets to the STRING network platform and set the protein species to “*Homo sapiens*” and the minimum interaction threshold to “medium confidence.” A protein-protein-interaction (PPI) network was then constructed, and the data list, in the form of a tab-separated value (TSV) file, was saved for further analysis.

### 2.5. The Screening of Key Active Compounds and Targets

The TSV data list was imported into Cytoscape, a bioinformatics image-processing software package, and a plug-in was used for the network topology analysis in an attempt to construct a direct or indirect compound or target protein network, thus revealing the effect of Yupingfeng powder for the prevention and treatment of NCP. We analyzed topological parameters such as “degree centrality,” “closeness centrality,” and “betweenness centrality” and selected nodes for which degree centrality was more than twofold higher than the median of the nodes. The selected nodes were then used as key active compounds and targets (hubs).

### 2.6. Analysis of the Biological Processes of Key Targets

Gene Ontology (GO) functional enrichment analysis was carried out by using the GOEAST analysis tool; this allowed us to describe the biological process, cellular components, and molecular functions of the gene products. In the present study, we evaluated biological process, cellular components, and molecular function according to statistical probability (*p*) and false discovery rate values. The annotated terms were then used to carry out cluster analysis, thus resulting in cluster scores: the higher the scores, the more important the biological processes regulated by the key genes.

### 2.7. KEGG Signaling Pathway Enrichment Analysis

Next, we analyzed the enrichment of KEGG signaling pathways for the key target genes using the DAVID tool. This analysis allowed us to identify the key signaling pathways of Yupingfeng powder when used to treat NCP. The DAVID database integrates various database resources, and the gene set was enriched and analyzed using an improved Fisher's exact test algorithm, in which *p* < 0.01 was regarded as the threshold for statistical significance with regard to KEGG pathway enrichment analysis. According to the functional items in the enriched pathway, we were able to identify potential targets for herbal medicine in related signaling pathways.

### 2.8. Molecular Docking

First, we downloaded mol2 files for kaempferol, quercetin, and wogonin from the PubChem database. After minimizing the energy of the downloaded compounds through Chem3D, the small molecule compounds were imported into MGLTools-1.5.6 software and processed to obtain pdbqt files. The 3CLpro (PDB ID: 6LU7, Resolution: 2.16 Å) crystal structure was downloaded in PDB format from the Protein Data Bank (PDB). After deleting irrelevant small molecules using Pymol2.1 software, the protein molecules were imported into MGLTools-1.5.6 software to delete water molecules, add hydrogen atoms, set the atom type, and calculate the protein charge; data were then saved as pdbqt files. All processed compounds were used as small-molecule ligands, and the protein targets were used as receptors. The center position of the Grid Box was determined according to the site of interaction for the small molecule and the target (*x* = −9.87, *y* = 12.982, *z* = 67.654), along with the length, width, and height (50 × 50 × 50). Finally, molecular docking was performed with AutoDock 4.2 software. The Lamarckian genetic algorithm was used for molecular docking calculations as follows: a population of 150, a maximum energy evaluation of 25 million, a maximum of 2000, a crossover rate of 0.8, and a mutation rate of 0.02. Independent docking was performed 100 times, and the final docking complex structure was evaluated based on the binding free energy. Molecular docking results were visualized by Pymol2.1 software. The action mode of the compound and the target protein was analyzed to identify the nature of the interaction between the compound and the protein residue, including hydrogen bond interaction, *π*-*π* interaction, and hydrophobic interaction. Then, by referring to the docking score of the compounds, we considered whether the compounds screened had similar activities to the positive compound.

### 2.9. Molecular Dynamic Simulation

Molecular dynamic (MD) simulations of protein and compound complexes were performed using Desmond version 2020. Here, the molecular force field in the MD simulation was selected as OPLS3e, and the TIP3 water model was used to solvate the system. Charges of the system were neutralized by adding ions. Energy minimization for the entire system was achieved using the OPLS3e force field (all-atomic force field). The geometric structure of water molecules, the bond lengths, and bond angles of heavy atoms were constrained by the SHAKE algorithm. The continuous system was simulated by applying periodic boundary conditions, and long-range static electricity was maintained using the particle grid Ewald method. An NPT ensemble with a temperature of 300 k and a pressure of 1.0 bar was used to balance the system. The Berendsen coupling algorithm was used for the coupling of temperature and pressure parameters. During the subsequent preparation of the system, a 100 ns operation was performed with a time step of 1.2 fs and a track record was performed every 10 ps, which recorded a total of 1000 frames. The root-mean-square deviation (RMSD) of the main chain atoms was calculated, and graphical analysis was performed to investigate the nature of the protein-ligand interaction.

## 3. Results

### 3.1. The Active Components of Yupingfeng Powder and Target Selection

The active parameter data, along with the absorption-, distribution-, metabolism-, and excretion-related parameter data pertaining to the herbs, were obtained by the interrogation of several TCM databases; duplicated material was deleted. Ultimately, 38 main active ingredients were identified, encompassing 17 *Astragalus*, 4 *Atractylodes macrocephalae*, and 18 fangfeng species. Of these, MOL000033 was found to be a common compound for *Astragalus* and *Atractylodes macrocephala*. Furthermore, 714 component-related target protein names were predicted. The names of the target proteins were standardized and corrected using the UniProt platform, and any duplicated information was removed. Following this process, 218 target proteins were identified. In addition, as different active compounds can act on the same target, and different targets may be associated with the same compound, the multicomponent, multitarget, and multilevel characteristics of the Chinese herbal medicines were fully confirmed, as detailed in Table 1.

### 3.2. Disease Targets

By retrieving NCP-related target genes from the DisGeNET and GeneCards databases, merging disease target genes from each database, and deleting duplicate targets, 298 NCP-related target genes were obtained.

### 3.3. A Database for Intersecting Drug-Disease Target Genes

**Figure 1:**
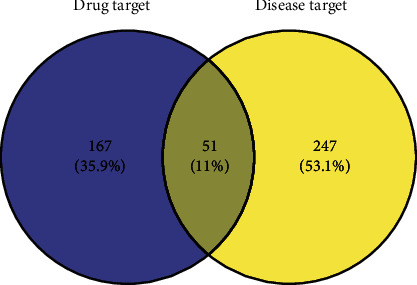
Venn diagram showing the potential NCP targets of Yupingfeng powder.

The predicted targets of the herbal medicines and disease target data were imported using the OmicShare online tool to construct a Venn diagram and identify intersections. Through this process, 51 common targets were obtained, comprising related targets of Yupingfeng powder acting on disease. As illustrated in Figure 1, the mapping rate of the target gene for Yupingfeng powder to NCP was 11%, thus suggesting that the prevention prescription would have a significant therapeutic effect in preventing and treating NCP.

### 3.4. A PPI Network for Shared Targets

**Figure 2:**
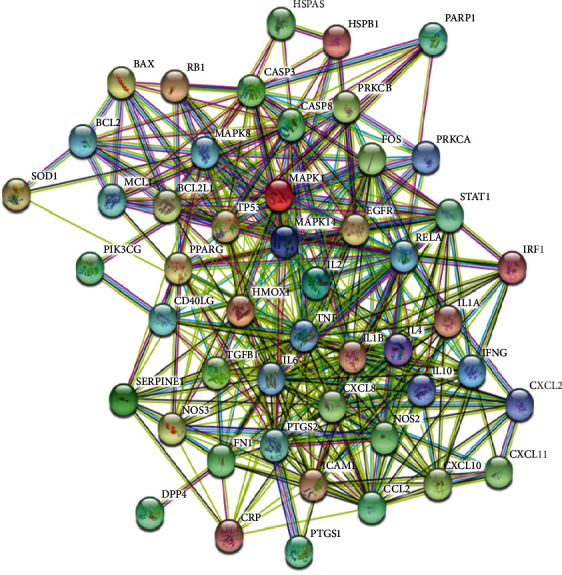
Network diagram of the core targets for Yupingfeng powder.

To better understand the mechanisms of Yupingfeng powder in relation to NCP, the 51 intersecting targets were imported into the STRING platform to generate a PPI network map and a TSV data list. There were 51 PPI targets in the network, and the 384 edges represent the PPI relationship. The mean node degree value of each node in the network was 15.1, and the mean local clustering coefficient was 0.641, as shown in Figure 2.

### 3.5. Key Compounds and Targets

The TSV list was imported into Cytoscape software, and network topology analysis was carried out. There were 49 nodes and 384 relationship edges in the network. Network topology analysis showed that the network density value was 0.055, the network concentration was 0.506, the network heterogeneity was 1.519, and the median value was 4.506. Then, we identified the key compounds and targets with nodes larger than 9.012. Note that the core node was used as the connection compounds or targets; thus, core nodes play an important role in the whole network. In our analysis, three key compounds and six key targets were identified, showing that Yupingfeng powder has great potential as a key compound or target and could play an important role in the prevention and control of NCP. Of these, the key compounds were MOL000098 (quercetin), MOL000173 (wogonin), and MOL000422 (kaempferol), which can interact with 62, 151, and 45 target proteins, respectively. The key targets were PTGS2, DPP4, PTGS1, NOS2, PPARG, and NOS3; these could interact with 32, 20, 17, 15, 12, and 10 compounds, respectively. These results indicate that there is synergism between the effective components of Yupingfeng powder and that the key targets also play a certain therapeutic role in the development of NCP, as detailed in Table 2.

### 3.6. GO Analysis of Biological Function Analysis

**Figure 3:**
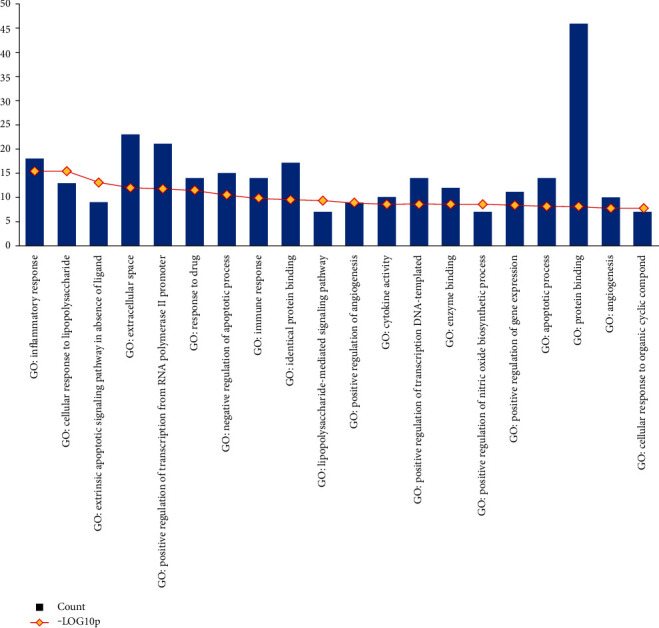
GO functional analysis.

The 51 intersecting targets were mapped to the GOEAST platform for enrichment analysis of the related biological processes, cellular components, and molecular functions. Using *p* < 0.01 as the significant enrichment screening standard, 82 functional enrichments were identified. Moreover, the first 20 major GO functional enrichment analysis results involved 15 significant enrichments to biological processes, encompassing inflammatory response, cellular response to lipopolysaccharide, extrinsic apoptotic signaling pathways in the absence of a ligand, positive regulation of transcription from the RNA polymerase II promoter, response to drugs, negative regulation of the apoptosis process, immune response, the lipopolysaccharide-mediated signaling pathway, positive regulation of angiogenesis, positive regulation of transcription, DNA-templated, positive regulation of nitric oxide biosynthesis, positive regulation of gene expression, angiogenesis, and cellular response to organic cyclic compounds. Significant enrichment was observed in relation to cell composition, mainly involving extracellular levels. There was also enrichment pertaining to several molecular functions, mainly involving identical protein binding, cytokine activity, enzyme binding, and protein binding. The results of GO enrichment analysis are depicted in Figure 3.

### 3.7. KEGG Pathway Analysis

**Figure 4:**
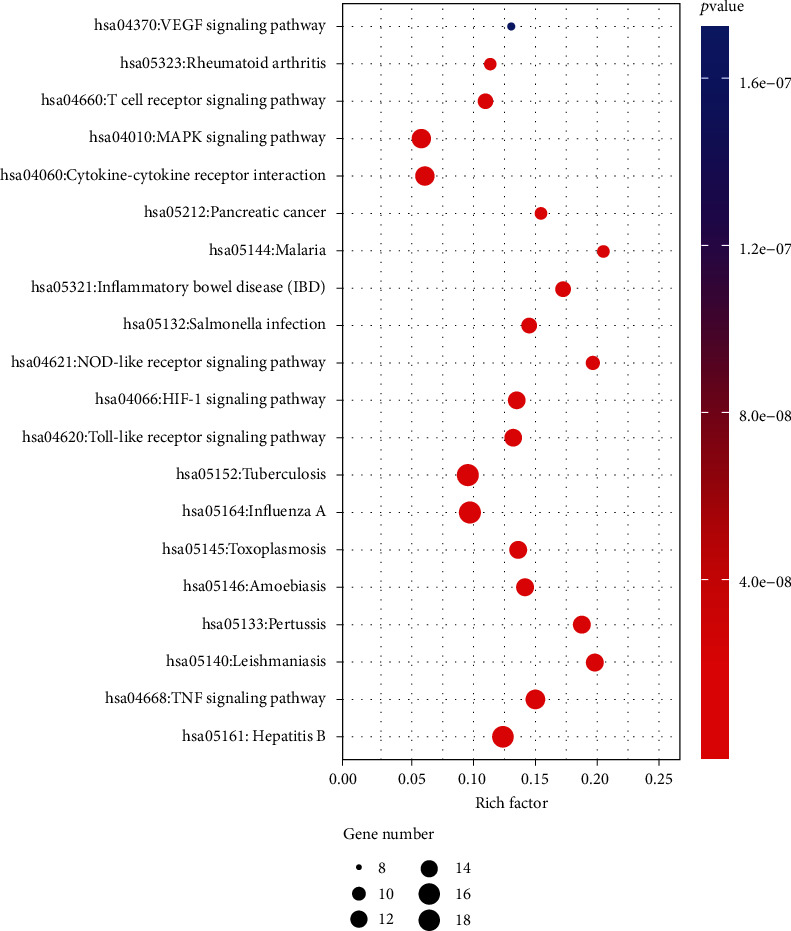
Bubble diagram showing the KEGG pathway analysis.

Next, the 51 intersecting targets were mapped to the DAVID tool for KEGG pathway analysis. With screening criteria of *p* < 0.01, the top 20 major enrichment signal pathways were encapsulated in a bubble chart. We found that 12 signaling pathways were involved in related diseases, while the other eight signal pathways involved TNF, Toll-like receptor, HIF-1, NOD-like receptor, cytokine–receptor interaction, MAPK, T cell receptor, and VEGF signaling pathways. All signaling pathways overlapped with the key target enrichments related to Yupingfeng powder. These results suggest that the effective components of Yupingfeng powder may act on multiple signaling pathways to treat NCP, a finding that suggests an important direction for future research on Yupingfeng powder in the prevention and treatment of NCP (see Figure 4).

### 3.8. Molecular Docking

**Figure 5:**
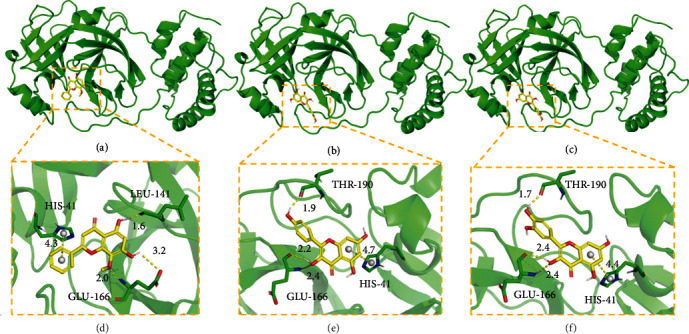
The binding mode of protein with compound. (a)–(c) The 3D structure for 3CLpro with wogonin, kaempferol, and quercetin. (d)–(f) The detail binding mode 3CLpro with wogonin, kaempferol, and quercetin. The backbone of protein was rendered in tube and colored in green. Compound is rendering by yellow. Yellow dash represents hydrogen bond distance or *π*-stacking.

The results of molecular docking are shown in Table 3 [9, 10, 11]. The complex of protein and small molecules was visually analyzed in Pymol2.1, as shown in Figure 5. In this experiment, kaempferol, quercetin, and wogonin were docked with the 3CLpro target protein. Molecular docking results showed that the compound and target protein exhibited good binding and a high degree of matching (Figures 5(b) and 5(c)). The binding energies were -7.75 kcal/mol, -7.47 kcal/mol, and -7.92 kcal/mol, respectively. Of these, wogonin showed an excellent docking score and binding mode with 3CLpro. The product from the docking of the compound and the protein was visualized using Pymol2.1 to identify the binding mode. The amino acid residues featured in the pocket of the compound and protein were clearly observed according to the binding mode. The active amino acid residues of the three compounds binding to the 3CLpro protein were HIS-41, LEU-141, GLU-166, and THR-190. The kaempferol, quercetin, and wogonin compounds were all flavonoid core compounds with high similarities in terms of binding mode (Figures 5(a)–5(c)). The binding modes of kaempferol and quercetin compounds with the 3CLpro protein were largely identical and formed strong hydrogen bond interactions with active sites in the protein (GLU-166 and THR-190; Figures 5(b) and 5(c)), thus resulting in short hydrogen bond distances and strong binding forces, thereby anchoring the small molecules in protein pockets. The wogonin compound had a slightly different binding mode with the protein, forming strong hydrogen bond interactions with the active site of the protein (GLU-166 and LEU-141; Figure 5(a)). The hydrogen bonds were shorter than that in the other two compounds and were relatively strong; this may be the main reason for the slightly better score. In addition, the three compounds could form a *π*-*π* conjugated interaction with HIS-41; this is important as it stabilized the small molecules in the pocket.

### 3.9. Molecular Dynamic Simulation Analysis

**Figure 6:**
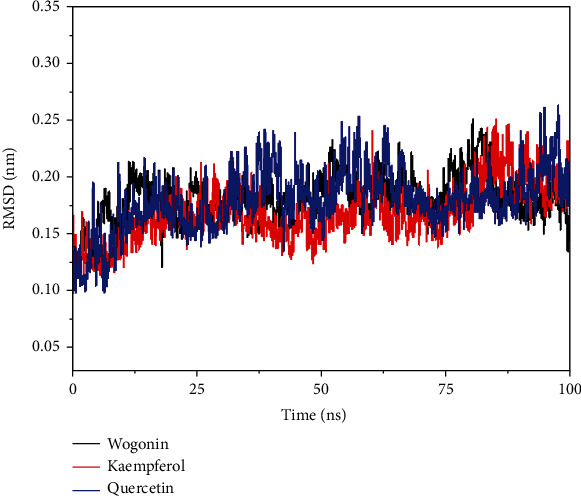
RMSD plot of molecular dynamic simulations between the protein and compounds.

**Figure 7:**
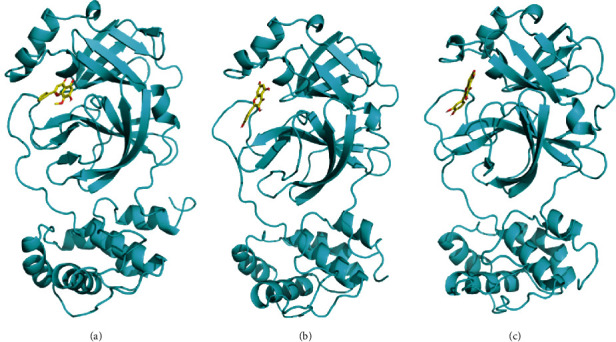
The 3D complex structure of the protein with compounds. (a)–(c) represent 3CLpro with wogonin, kaempferol, and quercetin, respectively.

To further study the interaction between small molecules and proteins, we used molecular dynamic simulations to perform 100 ns molecular dynamic operations on proteins and small molecule complexes. The relationship between RMSD and the stability of the reactive protein and small molecules was analyzed; the larger the RMSD, the more unstable the protein. As shown in Figure 6, small molecules fluctuated at the beginning and tended to stabilize during movement, which mainly reflected the continuous collisions between small molecules and active sites in the protein pocket. The RMSD of kaempferol, quercetin, wogonin, and the 3CLpro target proteins was small and reached equilibrium at about 15 ns, thus indicating a good combination of small molecules and proteins. Molecular dynamic simulations verified that small molecules could bind to the active sites of the receptor complex (Figures 7(a)–7(c)). During the simulated binding of kaempferol, quercetin, and wogonin to the 3CLpro target protein complex, the total binding free energy was -15.61 kcal/mol, -18.91 kcal/mol, and -15.49 kcal/mol, respectively. The Van der Waals force and electrostatic interactions contributed significantly to the binding process of small molecules and target proteins. The polar solvation energy inhibited the binding process. Wogonin had the largest polar solvation energy, mainly because it contains a methoxy group and significantly fewer hydroxyl groups than kaempferol and quercetin, thus directly enhancing its hydrophobicity and increasing its polar solvation energy. Comparison between the energy changes of the three compounds revealed that when forming a complex with the protein, the Van der Waals forces of wogonin contributed greater than electrostatic interactions due to its hydrophobicity. The kaempferol and quercetin compounds contained multiple hydroxyl groups and protein sites that could form multiple hydrogen bonds. Thus, electrostatic interaction is dominant. Molecular dynamic simulation of the three compounds also showed that significantly fewer hydrogen bonds were formed by wogonin and the active sites of the protein: only two main hydrogen bonds were formed with GLU-166 and LEU-141. In contrast, kaempferol and quercetin formed three hydrogen bonds with GLU-166, TYR-54, and GLN-192, see Tables 4–7 for details.

## 4. Discussion

At present, the world is dealing with the prevention and control of major pandemic. It is therefore particularly important to undertake comprehensive prevention and coordination measures to avoid pathogens, adjust diets, control emotions, strengthen bodies, and apply any other measures as necessary to achieve the optimum preventative effect. Yupingfeng powder is the basis of TCM prevention prescriptions issued in provinces and cities throughout China. Owing to its ability to deal with different syndromes and compatibility considerations, its diagnostic capability, and its flexibility with regard to individual consolations and treatment schemes, TCM has been found to achieve notable clinical outcomes in combating NCP. The first principle of TCM in pandemic prevention is to support and strengthen qi and to strengthen the surface of the body. This approach is not only in line with the underlying theory of TCM to treat a disease before it occurs but can also improve autoimmune function by supporting the healthy qi of the human body, so as to give full opportunity to the unique theoretical, practical, and herbal advantages of TCM. Thereby, TCM can be involved in the whole process of prevention, treatment, and prognosis related to the current pandemic in a multifaceted manner by strengthening the early preventative advantages of the uninfected and carrying out early detection and prognosis in the infected in isolation, thus allowing timely diagnosis and treatment. Thus, when combined with Western medicine, complementary TCM advantages should be provided to comprehensively improve the clinical efficacy of antipandemic strategies.

Through ancient herbal books, critical modern research, and evidence from animal experiments, it has been discovered that Yupingfeng powder has high clinical application value in the prevention of disease, immune regulation, and antiviral application [12, 13, 14]. In the present study, we performed biological enrichment analysis of the key active components and targets of Yupingfeng powder and found that the main enrichments related to the respiratory and immune systems. Our analysis suggested that these enrichments involved signaling pathways related to hepatitis B, pertussis, influenza A, tuberculosis, Salmonella infection, inflammatory bowel disease, rheumatoid arthritis, pancreatic cancer, and other diseases, thus suggesting that this prescription has a certain targeted regulatory role in the treatment of viral or bacterial infectious diseases and therefore has the potential to exert antibacterial and antiviral functionality. Furthermore, we found that the TNF, Toll-like receptor, HIF-1, NOD-like receptor, cytokine receptor interaction, MAPK, T cell receptor, VEGF, and other signaling pathways related to immune and inflammatory responses were all affected by Yupingfeng powder.

Currently, it is thought that TNF is mainly involved in cell apoptosis, cell proliferation, inflammation, and immune regulation. It has also been theorized that Yupingfeng powder may participate in the process of immune activation and regulation through the TNF signal pathway, thus affecting viral replication and persistence [15]. Toll-like receptors and NOD-like receptors not only act as extracellular and intracellular pattern-recognition receptors, respectively, but also stimulate the expression of different effector molecules through the cascade reaction of signal transduction. Moreover, they can jointly act on the innate immune process of infectious diseases, detect the invasion of pathogens, and initiate a protective response [16]. Research has also shown that, when the body is infected, it will induce different types of immune cells to generate physiological and pathological coordinated responses to effectively regulate cell proliferation, migration, differentiation, and other metabolic activities, in which HIF regulates a variety of effector molecules, signal transduction molecules, and transport molecules of cell metabolism. Consequently, the metabolism, differentiation, and immune function regulation of T cells will also be affected. In addition, the HIF-1*α*–VEGF-A axis plays an important role in regulating the immune and autoimmune functions of various pathogens. It has been postulated that Yupingfeng powder can build the immune microenvironment of the body, balance the immune system and eliminate inflammation, regulate the T cell receptor signal pathway, and enhance cell transformation ability to cope with the two-way regulatory mechanism responsible for immune enhancement and immunosuppression [17, 18, 19]. Moreover, the MAPK pathway is the main cell signaling system to be activated by a variety of viruses and also plays a key role in the regulation of cell proliferation, stress, inflammation, differentiation, transformation, apoptosis, and other processes. Accordingly, it has been suggested that the MAPK signaling pathway may be activated to induce a cytopathic effect and then play a role in inhibiting viral replication [20].

In this study, we conducted molecular docking experiments involving kaempferol, quercetin, and wogonin with the 3CLpro target protein. Analysis showed that the three compounds bound strongly with the 3CLpro target protein. To further study the mechanism of interaction between small molecules and protein pockets, we performed 100 ns molecular dynamic simulations on the three complexes. The simulation results showed that kaempferol, quercetin, and wogonin could form stable complexes with the 3CLpro protein. The contribution of active sites to the stability of small molecules was also analyzed. Our findings provide reference guidelines for future mechanistic research related to small molecules and proteins. However, our virtual screening results were mainly aimed at the theoretical level, thus warranting experimental pharmacological verification. Therefore, our future work will focus on the design, modification, and transformation of the compounds that bind to 3CLpro based on our knowledge of docking mode, the characteristics of the binding pocket in the receptor's crystal structure, and clinical trials. Collectively, these studies will further enhance the specificities of these medicines in regulating the biological activity of 3CLpro, thus creating new drugs with better specificities.

## 5. Conclusions

By applying relevant computer simulation calculation methods, we identified three key compounds (kaempferol, quercetin, and wogonin), six key targets (PTGS2, DPP4, PTGS1, NOS2, PPARG, and NOS3), and eight important signal pathways (TNF, Toll-like receptor, HIF-1, NOD-like receptor, cytokine receptor interaction, MAPK, T cell receptor, and VEGF signal pathway) involved in the action of Yupingfeng powder on NCP. We also identified that some signaling pathways were enriched and overlapped with the key targets related to Yupingfeng powder. Molecular dynamic simulations showed that kaempferol, quercetin, and wogonin could form stable complexes with the 3CLpro protein. This study not only clarified the efficacy of the mechanistic action of Yupingfeng powder; it also revealed the therapeutic effects of prescription drugs on NCP through multichannel and multiple approaches aimed towards intervention and regulation. It is hoped that this study will stimulate additional research concerning new targets and new pathways within the herbal medicine field, as well as further exploration of the mechanistic actions of various herbal medicines in respect of the observed synergistic effect, thereby providing innovation and facilitating the development of TCM. In the present work, the molecular mechanisms of a natural herbal medicine were investigated, and its complex mechanism was revealed at the molecular level, thus providing a theoretical basis for specific research and the development of natural and antiviral Chinese herbal medicines for clinical application. Furthermore, our findings confirm that the clinical application of TCM is recommended as the main entry point for prevention, to benefit from the proven advantages of both Chinese herbal medicine and Western medicine, and as a strategy for multidisciplinary combination therapy towards the prevention and control of NCP.

## Figures and Tables

**Table 1:** Main active component information of Yuping wind powder.

Herbs	TCMSP ID	Compound	OB	DL	Targets
Fangfeng	MOL011737	Divaricatacid	87	0.32	5
Huangqi	MOL000378	7-O-methylisomucronulatol	74.69	0.3	45
Huangqi	MOL000392	Formononetin	69.67	0.21	39
Huangqi	MOL000433	Folic acid	68.96	0.71	3
Fangfeng	MOL000011	(2R,3R)-3-(4-hydroxy-3-methoxy-phenyl)-5-methoxy-2-methylol-2,3-dihydropyrano[5,6-h][1,4]benzodioxin-9-one	68.83	0.66	18
Huangqi	MOL000380	(6aR,11aR)-9,10-dimethoxy-6a,11a-dihydro-6H-benzofurano[3,2-c]chromen-3-ol	64.26	0.42	22
Baizhu	MOL000022	14-acetyl-12-senecioyl-2E,8Z,10E-atractylentriol	63.37	0.3	1
Fangfeng	MOL011732	Anomalin	59.65	0.66	7
Huangqi	MOL000211	Mairin	55.38	0.78	1
Baizhu	MOL000049	3*β*-Acetoxyatractylone	54.07	0.22	16
Huangqi	MOL000371	3,9-Di-O-methylnissolin	53.74	0.48	23
Huangqi	MOL000239	Jaranol	50.83	0.29	13
Fangfeng	MOL011730	11-Hydroxy-sec-o-beta-d-glucosylhamaudol_qt	50.24	0.27	7
Huangqi	MOL000354	Isorhamnetin	49.6	0.31	35
Huangqi	MOL000439	Isomucronulatol-7,2′-di-O-glucosiole	49.28	0.62	1
Huangqi	MOL000417	Calycosin	47.75	0.24	22
Huangqi	MOL000098	Quercetin	46.43	0.28	151
Fangfeng	MOL001942	Isoimperatorin	45.46	0.23	1
Fangfeng	MOL011749	Phelloptorin	43.39	0.28	5
Fangfeng	MOL001494	Mandenol	42	0.19	3
Huangqi	MOL000422	Kaempferol	41.88	0.24	62
Fangfeng	MOL002644	Phellopterin	40.19	0.28	12
Fangfeng	MOL007514	Methyl icosa-11,14-dienoate	39.67	0.23	1
Fangfeng	MOL013077	Decursin	39.27	0.38	16
Huangqi	MOL000442	1,7-Dihydroxy-3,9-dimethoxy pterocarpene	39.05	0.48	4
Fangfeng	MOL011753	5-O-Methylvisamminol	37.99	0.25	24
Huangqi	MOL000296	Hederagenin	36.91	0.75	22
Fangfeng	MOL000359	Sitosterol	36.91	0.75	3
Fangfeng	MOL000358	Beta-sitosterol	36.91	0.75	37
Huangqi	MOL000379	9,10-Dimethoxypterocarpan-3-O-*β*-D-glucoside	36.74	0.92	3
Fangfeng	MOL003588	Prangenidin	36.31	0.22	15
Huangqi and Baizhu	MOL000033	(3S,8S,9S,10R,13R,14S,17R)-10,13-Dimethyl-17-[(2R,5S)-5-propan-2-yloctan-2-yl]-2,3,4,7,8,9,11,12,14,15,16,17-Dodecahydro-1H-cyclopenta[a]phenanthren-3-ol	36.23	0.78	1
Baizhu	MOL000072	8*β*-Ethoxy atractylenolide III	35.95	0.21	5
Fangfeng	MOL001941	Ammidin	34.55	0.22	8
Fangfeng	MOL011747	Ledebouriellol	32.05	0.51	14
Fangfeng	MOL011740	Divaricatol	31.65	0.38	16
Huangqi	MOL000387	Bifendate	31.1	0.67	7
Fangfeng	MOL000173	Wogonin	30.68	0.23	45

**Table 2:** Network topological characteristics of key compounds and targets.

Node	Node name	Degree centrality	Betweenness centrality	Closeness centrality
C17	Quercetin	47	0.588	0.656
PTGS2	Prostaglandin G/H synthetase 2	32	0.305	0.621
DPP4	Dipeptidyl peptidase IV	20	0.101	0.526
C32	Wogonin	19	0.111	0.463
C13	Kaempferol	18	0.075	0.453
PTGS1	Prostaglandin G/H synthetase 1	17	0.083	0.506
NOS2	Inducible nitric oxide synthetase 2	15	0.025	0.380
PPARG	Peroxisome proliferator activated receptor *γ*	12	0.026	0.461
NOS3	Endothelial nitric oxide synthetase	10	0.026	0.451

**Table 3:** The binding energy of key active compounds in Yupingfeng powder and 2019 ncov 3CL Hydrolase.

Target ID	Compounds	Structure	PubChem CID	CAS number	Docking score (kcal/mol)	Combination type
3CLpro	Kaempferol	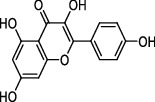	5280863	520-18-3	-7.75	Hydrogen bonds, Hydrophobic interactive
3CLpro	Quercetin	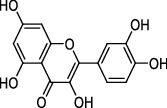	5280343	117-39-5	-7.47	Hydrogen bonds, Hydrophobic interactive
3CLpro	Wogonin	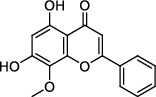	5281703	632-85-9	-7.92	Hydrogen bonds, Hydrophobic interactive

Note: Binding energy function [9–11]: ∆Gbind = C_lipo−lipo_∑f(T | r) + C_hbond−neuto−neut_∑g(∆r)h(∆*α*) + C_hbond−charged−charged_∑g(∆r)h(∆*α*) + C_max−metal−ion_∑f(T | m) + C_rotb_H_rotb_ + C_polar−phob_V_polar−phob_ + C_coul_E_coul_ + C_vdW_E_vdW_ + solvationerms. ∆Gbind: binding energy function; C_lipo-lipo_: the energy sum of the hydrophobic interaction; C_hbond-neuto-neut_: neutral hydrogen bond interactions; C_hbond-charged-charged_: the energy sum of charged hydrogen bond interactions; C_max-metal-ion_: the energy sum of the coordination action of metal ions; C_rotb_ H_rotb_: rotational bond energy; C_polar-phob_V_polar-phob_: polar interaction; C_coul_E_coul_: electrostatic interaction; C_vdW_*E*_vdW_: Van der Waals force; solvationerms: solvation energy.

**Table 4:** The analysis of binding energy for target with compound.

Energy type	Binding free energy/(kcal/mol)		
	Wogonin	Kaempferol	Quercetin
VDW	-27.15	-26.47	-29.63
Eele	-22.97	-28.28	-31.35
Egb	30.38	23.99	21.62
Esurf	-3.87	-4.11	-4.29
*Δ*G gas	-36.21	-35.97	-35.4
*Δ*G solv	25.23	24.23	20.23
*Δ*G total	-15.49	-15.61	-18.91

**Table 5:** Hydrogen bond interaction parameters for target with wogonin.

Atom	Residues	Bond distance/(Å)	Bond angle/(°)	Proportion/(%)
O3	MET-49	2.92	143.38	1.13
O5	LEU-141	2.90	145.23	36.48
O3	CYS-44	2.88	153.17	1.39
O3	GLN-192	2.87	140.11	0.97
O3	GLU-166	2.84	149.23	41.24
O5	LEU-167	2.71	150.01	1.48
O4	ASP-187	2.65	138.76	1.32

**Table 6:** Hydrogen bond interaction parameters for target with kaempferol.

Atom	Residues	Bond distance/(Å)	Bond angle/(°)	Proportion/(%)
O5	GLU-166	2.96	140.32	13.25
O3	ASP-187	2.86	148.25	1.20
O2	MET-49	2.85	154.14	1.38
O3	LEU-167	2.74	142.45	0.97
O2	THR-190	2.71	147.21	3.11
O6	CYS-44	2.63	143.52	1.15
O5	LEU-141	2.59	156.47	2.23
O6	TYR-54	2.48	150.59	35.43
O5	GLN-189	2.48	152.43	1.93
O4	GLN-192	2.37	139.78	35.22

**Table 7:** Hydrogen bond interaction parameters for target with quercetin.

Atom	Residues	Bond distance/(Å)	Bond angle/(°)	Proportion/(%)
O6	CYS-44	3.00	156.71	3.13
O7	GLN-189	2.86	153.58	2.78
O2	THR-190	2.84	147.11	3.25
O3	LEU-167	2.81	150.19	1.02
O2	MET-49	2.74	147.25	0.75
O7	LEU-141	2.74	160.24	1.09
O4	GLN-192	2.73	148.29	36.43
O7	ASP-187	2.71	140.32	0.87
O3	TYR-54	2.62	152.65	27.34
O5	GLU-166	2.59	154.21	15.76

## Data Availability

The data used to support this study can be obtained from the corresponding authors, according to the requirements of the journal. The data supporting the relevant research conclusions are openly available. Provided URLs or supplier details for all key software packages are as follows: TCMSP: A traditional Chinese medicine systems pharmacology database and analysis platform, available at http://www.tcmspw.com/tcmsp.php. ETCM: An encyclopedia of traditional Chinese medicine, available at http://www.nrc.ac.cn:9090/ETCM. TCMIP: A traditional Chinese medicine (including prescriptions) data-mining platform, available at http://www.tcmip.cn. UniProt platform: A database of protein sequence and functional information, available at https://www.uniprot.org. DisGeNET: A database of gene–disease associations, available at https://www.disgenet.org. GeneCards: A database of human genes that provides concise genomic-related information on all known and predicted human genes, available at https://www.genecards.org. OmicShare online tool: See https://www.omicshare.com/tools. STRING: STRING (Search Tool for the Retrieval of Interacting Genes/Proteins) is a biological database and web resource of known and predicted protein–protein interactions, available at https://string-db.org. Cytoscape software: See http://www.cytoscape.org/. GOEAST: GOEAST (Gene Ontology Enrichment Analysis Software Toolkit), available at http://omicslab.genetics.ac.cn/GOEAST. KEGG: KEGG (Kyoto Encyclopedia of Genes and Genomes) is a database resource for understanding high-level functions and utilities of the biological system, such as the cell, the organism, and the ecosystem, from molecular-level information, especially large-scale molecular data sets generated by genome sequencing and other high-throughput experimental technologies, see https://www.kegg.jp. DAVID: DAVID (Database for Annotation, Visualization, and Integrated Discovery) bioinformatic microarray analysis, available at https://david.ncifcrf.gov. PubChem: See https://pubchem.ncbi.nlm.nih.gov. MGL Tool software: Software developed at the Molecular Graphics Laboratory (MGL) of the Scripps Research Institute for visualization and analysis of molecular structures, available at http://mgltools.scripps.edu. PDB: RCSB (Research Collaboratory for Structural Bioinformatics) Protein Data Bank, available at https://www.rcsb.org. AutoDock software: A molecular docking and virtual screening program, available at http://vina.scripps.edu. Pymol software: A molecular visualization system, available at https://pymol.org.

## Abbreviations

BC: Betweenness centrality
COVID-19: Coronavirus disease 2019
CC: Closeness centrality
DC: Degree centrality
DL: Drug likeness
ETCM: The encyclopedia of traditional Chinese medicine database
GO: Gene Ontology
HIF-1: Hypoxia-inducible factor 1
KEGG: The Kyoto Encyclopedia of Genes and Genomes
MAPK3: Mitogen-activated protein kinase 3
MAPK8: Mitogen-activated protein kinase 8
NCP: Novel coronavirus pneumonia
OB: Oral bioavailability
PPI: Protein-protein interaction network
SARS-CoV-2: Severe acute respiratory syndrome coronavirus 2
TCM: Traditional Chinese medicine
TCMSP: Traditional Chinese medicine systems pharmacology database and analysis platform
TCMIP: Integrative pharmacology-based research platform of traditional Chinese medicine
TNF: Tumor necrosis factor
VEGF: Vascular endothelial growth factor.
